# Twenty Propositions on Milk-Sickness

**Published:** 1857-11

**Authors:** J. W. Crooks

**Affiliations:** Rockport, Ind., in a Letter to one of the Editors


ARTICLE II.—*Twenty Propositions on Milk-Sickness.* By J. W. CROOK, M.D., of Rockport, Ind., in a Letter to one of the Editors.

DEAR DOCTOR,—In compliance with request, I proceed to furnish for your inspection and supervision a few observations upon the malady in question, as they have presented themselves in my practice. I shall present, in a condensed form, conclusions drawn from observation and experience, for near twenty years, without giving various reasons for the same, as it would require considerable space, and besides, in this as in other diseases, many *facts* exist that are not readily and satisfactorily explicable.

1st. The disease known as "Milk-Sickness" is not of malarious origin, nor in the least degree influenced by this agent.

2d. It is of less frequency in miasmal districts in this county than any other locality, occurring almost universally in the high land comparatively exempt from miasmal diseases.

3d. Depends upon an unknown poison taken into the system of the animal, thence transmitted to the human species through the medium of meat, milk or butter.

4th. It most usually, if not *always*, occurs early in the spring when pasturage is scarce, and late in the fall when forest grazing is pretty well over. It is prevailing at the present time, amongst the stock of this county, to a greater degree than for a number of years past.

5th. In no instance has it ever been known in this county during any other than the grazing season, and then *only* upon lands that had never been in cultivation.

6th. A late and wet spring—a dry summer, followed by a wet fall—favors its development among stock.

7th. It seems that the circumstances favoring a rapid and succulent growth with a moderate scarcity of grass, and a kind of necessity for stock eating most anything that is at all to be tolerated by their systems, are exigencies conducive to the "Trembles."

8th. Hence, I have come to the conclusion that this malady is induced in cattle, horses, hogs, sheep, etc., by them eating some poisonous vegetable.

9th. Under the assumed principle that the poisonous property depends upon some unknown mineral exhalation from the earth, or other floating noxious quality in the atmosphere, there is no good reason why it should not affect animals in the barren barn yard, the cultivated pastures, stubble fields, or the human species in his domicil.

10th. It was the opinion of the lamented Dr. Drake, that the disease was caused by cattle eating a species of the poison oak (Rus. Toxicodendron), and from observations incited by reading his paper, I am *strongly* of this opinion.

11th. I have inspected, time and again, localities, wood pastures, etc., where sheep, cows and horses had contracted the Trembles, and in *no* instance was this vegetable found wanting.

12th. I have often gone into limited pastures and found the stub of the vine where the leaves and tops had been eaten off, the calf down with the Trembles, and patients at the house with the Milk-Sick.

13th. An intelligent farmer saw a sheep eating freely of this vegetable, from a luxuriant growth that had sprung up along side of a decaying log; the identical sheep died next day of Trembles.

14th. Stock are not passionately fond of this weed, though they will eat it in times of general scarcity in proportion to the requirements of their appetites.

15th. The poison oak is peculiar to high broken grounds, and scarcely, if ever, found in alluvial bottoms, at least not in this county. The disease is never contracted in the bottoms, in no instance of the kind. The people use beef, milk and butter without the least fear, and enjoy perfect immunity.

16th. It is certain that the slightest culture of the soil destroys the cause, and has never been known to re-appear; and it is equally certain and well known, that the Rus. Tox. answers to these peculiarities. It is but slightly attached to the earth, running along on the surface just under the leaves and other trash, throwing down fragile tendrils which attaches to the loose soil, and a weight of five pounds will break up the largest bunches.

17th. I have never known a case of the disease, or anything approximating its peculiarities, that was not traceable to the use of meat, milk or butter. Once, however, I had like to have been *stumped* over this position. A gentleman in town was attacked with *all* the prominent symptoms, but assured me there was no possible chance for Milk-sick; though using butter, it was from the most reliable source. He was treated as we ordinarily treat the disease, and finally got well. It was subsequently ascertained that although the cows were "kept up," it was in a sort of mixed pasture, in which woodland ground, that had never been in cultivation, predominated, and in that part which had been cultivated, there was but little for the cows to eat. I said my patient got well, yet he never felt fully *normal;* whenever the pupil of his eye covered the butter dealer, he exhibited slight evidences of the *trembles*, notwithstanding his proverbial good nature.

18th. The prominent symptoms of the disease consist of excessive nausea, vomiting first the ordinary contents of the stomach, bile, and such liquids as may have been swallowed; a glariry, tenacious mucous, very acrid and sour; and, finally, matter of a coffee-grown appearance; burning sensation in the stomach; excessive prostration; anxious and contracted countenance; leaden color and cool skin; *obstinate constipation;* with others that might be enumerated, with which no one is more familiar than yourself.

19th. The pathological condition is no doubt a peculiar gastritis, involving the entire inner portion of the stomach, but more particularly the pyloric region. I have not had the advantages of post mortem examinations. If you would not consider it vain, I would say that my opportunities for this kind of investigation have been very limited; for fifteen years past, I have not let a patient *die* of this disease! (Desp. Dark).

20th. Treatment. Well, it is not necessary to mention the indications to be fulfilled—these are apparent. And it is almost as unnecessary to note the treatment, for there is little or no difference now-a-days with the posted part of the profession.

To arrest the vomiting, I use morphine, blisters and alcoholic stimulants; and if I had to choose but one remedy, and use one only, I verily believe I would take the whiskey. I do not offer any reason or modus operandi, for or as to the whys or the wherefores; I simply *know* that whiskey is the thing in Milk-Sick, or in any other animal poison, as snakes' and bee stings, spider bites; and if I had a patient with hydrophobia, I would give him *whiskey* until he either lived or died. Don't make up your mind from this that I am *passionately* fond of the "critter," and am in danger from excessive use—I am only so under the circumstances detailed. I believe the alcoholic stimulant operates by a neutralizing influence upon the poison in the blood, and this influence, to a considerable extent, is mutual, as it is almost impossible to intoxicate the patient while under the poisonous influence.

As a purgative, I formerly gave calomel and castor oil; have not given it, however, for several years; mostly use ep. salts, combined with magnesia cal.

There is little or no difficulty in procuring the requisite action on the bowels, after quieting the stomach, and this once effected, the patient is about well.

Convalescence is usually rapid—relapses very rare; tonics, as Columbo bitters, or something of the kind, with generous diet, is all that is necessary.

